# Resolving species boundaries in the *Atlanta brunnea* species group (Gastropoda, Pterotracheoidea)

**DOI:** 10.3897/zookeys.899.38892

**Published:** 2019-12-12

**Authors:** Deborah Wall-Palmer, Mona Hegmann, Erica Goetze, Katja T.C.A. Peijnenburg

**Affiliations:** 1 Marine Biodiversity Group, Naturalis Biodiversity Center, P.O. Box 9517, 2300 RA Leiden, The Netherlands Naturalis Biodiversity Center Leiden Netherlands; 2 Institute for Biosciences, University of Rostock, Albert Einstein Straβe 3, 18059 Rostock, Germany University of Rostock Rostock Germany; 3 Department of Oceanography, University of Hawaii at Manoa, 1000 Pope Road, Honolulu, Hawaii, 96822, USA University of Hawaii at Manoa Hawaii United States of America; 4 Institute for Biodiversity and Ecosystem Dynamics (IBED), University of Amsterdam, P. O. Box 942480 1090 GE Amsterdam, The Netherlands University of Amsterdam Amsterdam Netherlands

**Keywords:** Atlantidae, biogeography, phylogenetic analysis, shell morphology, South Pacific Ocean

## Abstract

Atlantid heteropods are a family of holoplanktonic marine gastropods that occur primarily in tropical and subtropical latitudes. Atlantids bear a delicate aragonitic shell (<14 mm) and live in the upper ocean, where ocean acidification and ocean warming have a pronounced effect. Therefore, atlantids are likely to be sensitive to these ocean changes. However, we lack sufficiently detailed information on atlantid taxonomy and biogeography, which is needed to gain a deeper understanding of the consequences of a changing ocean. To date, atlantid taxonomy has mainly relied on morphometrics and shell ornamentation, but recent molecular work has highlighted hidden diversity. This study uses an integrated approach in a global analysis of biogeography, variation in shell morphology and molecular phylogenies based on three genes (CO1, 28S and 18S) to resolve the species boundaries within the *Atlanta brunnea* group. Results identify a new species, *Atlanta vanderspoeli*, from the Equatorial and South Pacific Ocean, and suggest that individuals of *A. brunnea* living in the Atlantic Ocean are an incipient species. Our results provide an important advance in atlantid taxonomy and will enable identification of these species in future studies of living and fossil plankton.

## Introduction

The heteropods (Pterotracheoidea) are a superfamily of marine gastropods (Order Littorinimorpha) consisting of three families: Pterotracheidae, Carinariidae and Atlantidae (Lalli and Gilmer 1989; Bouchet et al. 2017). While most marine gastropods are benthic in habitat, heteropods are holoplanktonic and live entirely in the upper water column. This study focuses on the Atlantidae (atlantids), the most species-rich family of heteropods, including more than 60 % of all heteropod species recognised so far (Seapy 1990; Wall-Palmer et al. 2018b). Atlantids are widespread and occur primarily in the epipelagic zone between the surface and 250 m, in tropical and subtropical latitudes (Wall-Palmer et al. 2018a). They are highly modified for life in the open ocean with several striking morphological adaptions such as the development of complex image-forming eyes, a foot that has adapted into a single swimming fin, and the reduction in size (< 14 mm) and weight of the laterally compressed, thin-walled (3–40 µm) shell (Lalli and Gilmer 1989). The entire shell is moved back and forth rapidly during swimming, so that the swimming fin and shell work together as dissimilar paired swimming appendages (Karakas et al. 2018). This locomotory design means that a structurally sound shell is vital for the survival of atlantids because it is necessary for all swimming, including capturing prey and evading predators. The shell of most atlantids is composed of aragonite, an unstable polymorph of calcium carbonate that is 50 % more soluble in the oceans than calcite (Sun et al. 2015). The chemical composition of the shell and persistent exposure to increasingly acidified waters in the upper water column makes atlantids likely to be particularly sensitive to ocean acidification (Wall-Palmer et al. 2016). However, sensitivity to ocean change is known to be species-specific in holoplanktonic marine gastropods. Roberts et al. (2014) found that different forms of the shelled pteropod *Limacina helicina* (Phipps, 1774) show contrasting trends in shell weight when exposed to undersaturation of calcium carbonate. Therefore, to gain deeper understanding of the consequences of a changing ocean, it is extremely important to resolve the species boundaries of holoplanktonic gastropods accurately, so that species-resolved environmental sensitivities can be determined.

To date, the taxonomy of atlantid heteropods has mainly relied on morphometrics and shell ornamentation, even though it is hard to tell some species apart only by use of morphological characteristics (Richter 1973). In particular, identification within the genus *Atlanta* Lesueur, 1817 is difficult, due to the similarities in body and shell forms (Ossenbrügger 2010). Additionally, there can be variation in morphology and ornamentation within a single species (e.g. *Atlanta selvagensis* de Vera & Seapy, 2006, Wall-Palmer et al. 2018b). Consequently, Richter (1973) suggested that morphometrics alone were insufficient to describe a new species, and assumed that as a result the number of heteropod species was largely underestimated. This underestimated diversity was confirmed in the first molecular phylogenetic study to include all atlantid morphospecies which found 30 % more mitochondrial cytochrome *c* oxidase subunit 1 (CO1) clades than there are described morphospecies (Wall-Palmer et al. 2018b). These authors found at least 10 additional atlantid clades that may represent new species in addition to the 23 currently described present-day species. This study, as well as many others (Goetze 2010; Burridge et al. 2015, 2019; Barco et al. 2016; Bode et al. 2017; Cornils et al. 2017), highlights the importance of combining morphology (morphometrics), biogeography and molecular analyses when exploring species diversity in the open ocean.

Based on morphological characters (shell, eye type, radula, operculum), atlantid heteropods are divided into groups of closely related species (Tesch 1949). The *Atlanta brunnea* group was first described as the *Atlanta turriculata* group by Tesch (1908), and prior to this study contained two species: *A. turriculata* d’Orbigny, 1836 and *A. brunnea* Gray, 1850 (= *Atlanta fusca* Souleyet, 1852). Additional diversity within this group has been recognised in morphological studies (Souleyet 1852; Tesch 1906; Richter 1961; van der Spoel 1972, 1976) and a single molecular study (Wall-Palmer et al. 2018b); however, the taxonomy of this group has not been investigated in sufficient detail. Here we use a combination of the morphological, biological/biogeographical and phylogenetic species concepts (De Queiroz 2007) to investigate the species boundaries within the *A. brunnea* group. We formally describe the previously recognised *A. turriculata* form B (van der Spoel 1972, 1976), also previously named *A. brunnea* form B (Wall-Palmer et al. 2018b) as *Atlanta vanderspoeli* sp. nov. named after Professor Siebrecht van der Spoel who first recognised *A. vanderspoeli* but did not describe it as a new species (Van der Spoel 1972, 1976).

## Materials and methods

### Specimen collection

**Figure 1.**
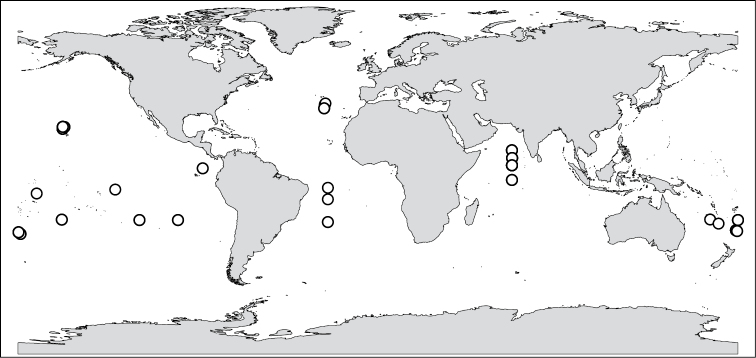
Collection locations for *A. brunnea* group specimens analysed in this study. Members of this species group are known to inhabit all oceans from 40N to 30S (Wall-Palmer et al. 2018b).

A total of 22 *A. brunnea*, 14 *A. vanderspoeli* (based on the descriptions of van der Spoel 1976) and 55 *A. turriculata* were examined in this study. All specimens have biogeographical data and have been analysed in at least one additional way (molecular, morphological or both, Table 1, Figure 1). Specimens were collected during several research cruises (Table 1) using various plankton nets. Collection methods have been described for all research cruises (Schmidt 1932; Hirai et al. 2015; Burridge et al. 2017a; Wall-Palmer et al. 2018b; Wall-Palmer et al. in preparation). Specimens from the DANA cruise of 1921 (two *A. brunnea*, two *A. vanderspoeli*, and two *A. turriculata*) were kindly made available by the Natural History Museum of Denmark (NHMD). All biogeographical data have been visualised using the software QGIS (QGIS Development Team 2016).

**Table 1.** Collection information for specimens examined in this study, with inclusion into each type of analysis as indicated at right. Specimens used for the combined gene phylogeny are indicated with +.

Species	BOLD/GenBank accession number	Museum accession number	Ocean	Cruise	Station	Biogeography Latitude/Longitude	Morphological analysis	Molecular analysis		
								CO1	28S	18S
*Atlanta brunnea*	AGD001-17	RMNH.MOL.341299	Atlantic	AMT24	05	34.75, -26.62		✓	✓	✓
	ATCP003-19	RMNH.MOL.341308	Atlantic	AMT27	09	35.30, -26.28		✓	✓	✓
	ATCP008-19	RMNH.MOL.341314	Atlantic	AMT27	11	32.87, -26.91	✓	✓	✓	✓
	ATCP009-19	RMNH.MOL.341315	Atlantic	AMT27	11	32.87, -26.91	✓	✓+	✓+	✓+
	ATCP010-19	RMNH.MOL.341316	Atlantic	AMT27	11	32.87, -26.91		✓+	✓+	✓+
	ATCP004-19	RMNH.MOL.341309	Atlantic	AMT27	37	-6.87, -25.04		✓		
	ATCP006-19	RMNH.MOL.341311	Atlantic	AMT27	41	-12.63, -25.05		✓		✓
	ATCP007-19	RMNH.MOL.341312	Atlantic	AMT27	47	-24.01, -25.05		✓+	✓+	✓+
	ATCP011-19	RMNH.MOL.341317	Atlantic	AMT27	47	-24.01, -25.05		✓+	✓+	✓+
	AGD008-17	RMNH.MOL.341304	Indian	SN105	04	8.02, 67.08		✓+	✓+	✓+
	AGD009-17	RMNH.MOL.341305	Indian	SN105	04	8.02, 67.08		✓		
	AGD010-17	RMNH.MOL.341300	Indian	SN105	08	4.38, 67.00		✓		
	AGD011-17	RMNH.MOL.341301	Indian	SN105	08	4.38, 67.00		✓+	✓+	✓+
	AGD012-17	RMNH.MOL.341302	Indian	SN105	08	4.38, 67.00		✓		
	AGD013-17	RMNH.MOL.341303	Indian	SN105	08	4.38, 67.00		✓		
	–	–	Indian	SN105	08	4.38, 67.00	✓			
	AGD002-17	RMNH.MOL.341313	Pacific	KH1110	05	-23.00, 180.01		✓	✓	
	ATCP001-19	RMNH.MOL.341306	Pacific	KOK1703	07	23.62, -157.61		✓	✓	
	ATCP002-19	RMNH.MOL.341307	Pacific	KOK1703	07	23.62, -157.61		✓	✓	
	ATCP005-19	RMNH.MOL.341310	Pacific	SO255	100	-28.52, 179.59		✓	✓	
	–	NHMD-232129	Pacific	DANA	3556 VIII	2.87, -87.63	✓			
	–	NHMD-232129	Pacific	DANA	3556 VIII	2.87, -87.63	✓			
*Atlanta vanderspoeli*	AGD003-17	RMNH.MOL.341320	Pacific	KH1110	15	-23.00, -119.27		✓		
	AGD004-17	RMNH.MOL.341321	Pacific	KH1110	15	-23.00, -119.27		✓	✓	✓
	AGD005-17	RMNH.MOL.341322	Pacific	KH1110	15	-23.00, -119.27		✓	✓	✓
	AGD006-17	RMNH.MOL.341323	Pacific	KH1110	21	-23.00, -100.00		✓		✓
	ATCP013-19	RMNH.MOL.341324	Pacific	KH1110	21	-23.00, -100.00		✓	✓	
	AGD007-17	RMNH.MOL.341325	Pacific	KH1110	21	-23.00, -100.00		✓	✓	✓
	ATCP012-19	RMNH.MOL.341319	Pacific	SO255	057	-29.95, -178.73	✓	✓		
	ATCP014-19	RMNH.MOL.341326	Pacific	SO255	057	-29.95, -178.73		✓		
	–	–	Pacific	SO255	073	-28.13, 179.02	✓			
	ATCP015-19	RMNH.MOL.341327	Pacific	SO255	073	-28.13, 179.02	✓	✓+	✓+	✓+
	ATCP016-19	RMNH.MOL.341328	Pacific	SO255	080	-29.10, -179.72	✓	✓+	✓+	✓+
	ATCP017-19	RMNH.MOL.341329	Pacific	SO255	080	-29.10, -179.72	✓	✓		
	–	NHMD-232153	Pacific	DANA	3613 V	-22.72, 166.10	✓			
	–	NHMD-232154	Pacific	DANA	3620 IV	-24.78, 170.31	✓			
*Atlanta helicinoidea*	ATCP052-19	RMNH.MOL.341459	Pacific	KOK1703	06	23.52, -156.78		✓+	✓+	✓+
	ATCP049-19	RMNH.MOL.341456	Pacific	KOK1703	08	23.62, -157.61		✓+	✓+	✓+
	ATCP047-19	RMNH.MOL.341454	Pacific	KOK1703	08	23.62, -157.61		✓+	✓+	✓+
*Atlanta turriculata*	AGD372-17	RMNH.MOL.341775	Indian	SN105	01	11.89, 66.97		✓		
	AGD367-17	RMNH.MOL.341779	Indian	SN105	01	11.89, 66.97		✓	✓	✓
	AGD368-17	RMNH.MOL.341780	Indian	SN105	01	11.89, 66.97		✓		
	AGD369-17	RMNH.MOL.341781	Indian	SN105	01	11.89, 66.97		✓		
	AGD370-17	RMNH.MOL.341782	Indian	SN105	01	11.89, 66.97		✓		
	AGD371-17	RMNH.MOL.341783	Indian	SN105	01	11.89, 66.97		✓		
	AGD376-17	RMNH.MOL.341774	Indian	SN105	04	8.02, 67.08		✓		
*Atlanta turriculata*	AGD375-17	RMNH.MOL.341776	Indian	SN105	04	8.02, 67.08		✓		
	AGD373-17	RMNH.MOL.341784	Indian	SN105	04	8.02, 67.08		✓+	✓+	✓+
	AGD374-17	RMNH.MOL.341785	Indian	SN105	04	8.02, 67.08		✓		
	AGD380-17	RMNH.MOL.341769	Indian	SN105	08	4.38, 67.00		✓		
	AGD378-17	RMNH.MOL.341777	Indian	SN105	08	4.38, 67.00		✓		
	AGD379-17	RMNH.MOL.341778	Indian	SN105	08	4.38, 67.00		✓		
	AGD377-17	RMNH.MOL.341786	Indian	SN105	08	4.38, 67.00		✓		
	ATCP123-19	RMNH.MOL.341814	Indian	SN105	08	4.38, 67.00	✓	✓	✓	✓
	AGD381-17	RMNH.MOL.341770	Indian	SN105	19	-2.95, 66.99		✓+	✓+	✓+
	AGD382-17	RMNH.MOL.341771	Indian	SN105	19	-2.95, 66.99		✓	✓	✓
	AGD383-17	RMNH.MOL.341772	Indian	SN105	19	-2.95, 66.99		✓		
	AGD384-17	RMNH.MOL.341773	Indian	SN105	19	-2.95, 66.99		✓		
	AGD363-17	RMNH.MOL.341797	Pacific	KH1110	05	-23.00, 180.01		✓	✓	✓
	AGD361-17	RMNH.MOL.341801	Pacific	KH1110	05	-23.00, 180.01		✓		
	AGD362-17	RMNH.MOL.341802	Pacific	KH1110	05	-23.00, 180.01		✓	✓	✓
	AGD365-17	RMNH.MOL.341798	Pacific	KH1110	08	-22.79, -158.10		✓		
	AGD364-17	RMNH.MOL.341799	Pacific	KH1110	08	-22.79, -158.10		✓		
	AGD366-17	RMNH.MOL.341800	Pacific	KH1110	08	-22.79, -158.10		✓		
	ATCP103-19	RMNH.MOL.341788	Pacific	KOK1703	01	22.91, -157.72		✓	✓	✓
	ATCP112-19	RMNH.MOL.341803	Pacific	KOK1703	01	22.91, -157.72	✓	✓	✓	
	ATCP102-19	RMNH.MOL.341787	Pacific	KOK1703	03	22.65, -157.69		✓	✓	
	ATCP116-19	RMNH.MOL.341807	Pacific	KOK1703	03	22.65, -157.69	✓	✓	✓	
	ATCP118-19	RMNH.MOL.341809	Pacific	KOK1703	03	22.65, -157.69	✓	✓	✓	
	ATCP120-19	RMNH.MOL.341811	Pacific	KOK1703	03	22.65, -157.69	✓	✓+	✓+	✓+
	ATCP122-19	RMNH.MOL.341813	Pacific	KOK1703	03	22.65, -157.69	✓	✓		
	ATCP124-19	RMNH.MOL.341815	Pacific	KOK1703	03	22.65, -157.69	✓	✓	✓	
	ATCP104-19	RMNH.MOL.341789	Pacific	KOK1703	05	22.65, -157.69		✓	✓	
	ATCP125-19	RMNH.MOL.341816	Pacific	KOK1703	05	22.65, -157.69	✓	✓	✓	✓
	ATCP127-19	RMNH.MOL.341818	Pacific	KOK1703	05	22.65, -157.69	✓	✓	✓	✓
	ATCP129-19	RMNH.MOL.341820	Pacific	KOK1703	05	22.65, -157.69	✓	✓	✓	
	ATCP113-19	RMNH.MOL.341804	Pacific	KOK1703	06	23.52, -156.78	✓	✓		
	ATCP117-19	RMNH.MOL.341808	Pacific	KOK1703	06	23.52, -156.78	✓	✓	✓	✓
	ATCP119-19	RMNH.MOL.341810	Pacific	KOK1703	07	23.62, -157.61	✓	✓	✓	✓
	ATCP115-19	RMNH.MOL.341806	Pacific	KOK1703	08	23.62, -157.61		✓	✓	✓
	ATCP121-19	RMNH.MOL.341812	Pacific	KOK1703	08	23.62, -157.61	✓	✓+	✓+	✓+
	ATCP105-19	RMNH.MOL.341790	Pacific	SO255	057	-29.95, -178.73		✓	✓	✓
	ATCP106-19	RMNH.MOL.341791	Pacific	SO255	073	-28.13, 179.02		✓+	✓+	✓+
	ATCP114-19	RMNH.MOL.341805	Pacific	SO255	073	-28.13, 179.02		✓		✓
	–	–	Pacific	SO255	073	-28.13, 179.02	✓			
	ATCP126-19	RMNH.MOL.341817	Pacific	SO255	073	-28.13, 179.02	✓	✓+	✓+	✓+
	ATCP128-19	RMNH.MOL.341819	Pacific	SO255	073	-28.13, 179.02	✓	✓	✓	✓
	ATCP107-19	RMNH.MOL.341792	Pacific	SO255	080	-29.10, -179.72		✓	✓	✓
	ATCP108-19	RMNH.MOL.341793	Pacific	SO255	080	-29.10, -179.72		✓	✓	✓
	ATCP109-19	RMNH.MOL.341794	Pacific	SO255	080	-29.10, -179.72		✓		✓
	ATCP110-19	RMNH.MOL.341795	Pacific	SO255	080	-29.10, -179.72		✓	✓	✓
	ATCP111-19	RMNH.MOL.341796	Pacific	SO255	100	-28.52, 179.59		✓		
	–	NHMD-232140	Pacific	DANA	3563 IV	-7.76, -131.37	✓			
	–	NHMD-232145	Pacific	DANA	3586 VII	-9.72, -170.67	✓			

### Imaging and morphological analysis

Specimens were identified based on the species keys of Richter and Seapy (1999), Seapy et al. (2003) and van der Spoel (1976), and imaged using a Zeiss automated z-stage light microscope at Naturalis Biodiversity Center, Leiden. Specimen shells were destroyed during the process of DNA extraction, so archived images provide the opportunity to verify in cases of uncertainty between morphologically identified specimens and molecular results. All images are available through the Barcode of Life Data System (BOLD; http://www.boldsystems.org) and are linked to physical DNA extracts at Naturalis Biodiversity Center (Table 1).

**Figure 2.**
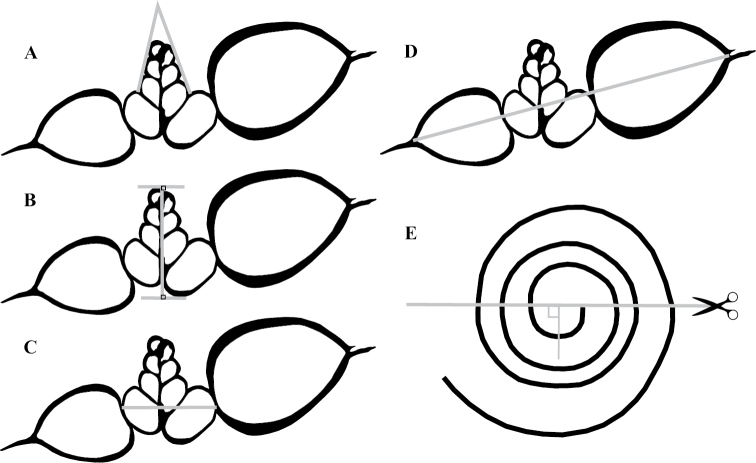
Examples of shell parameters measured from 2D slices of micro-CT scans. Measurements include **A** apical angle **B** larval shell height **C** maximum larval shell width, and **D** maximum adult shell diameter. The position of the slice through the 3D model to create a 2D image is shown in **E** relative to the suture.

Due to the small size of atlantid shells it is difficult to orientate them in a reproducible way for reliable morphometric measurements. Therefore, micro-computed tomography (micro-CT) was used to generate 3D-models for a subset of specimens in order to create 2D-slices through the larval shell for reproducible morphological measurements. A total of five *A. brunnea*, seven *A. vanderspoeli*, and 19 *A. turriculata* were imaged using a SkyScan 1172 high resolution micro-CT scanner. In total 958 images were collected per specimen using the following parameters: no filter, medium resolution camera, an image pixel size of 1.31–2.23 µm, a source voltage of 57 kV, a source current of 177 µA and an exposure time 600–800 ms. The rotation pitch was 0.2°, with averaging frames of 4, and random movement of 10. The software Avizo 9.0 was used to generate a 3D-surface of each shell, and to make 2D-slices perpendicularly through the earliest part of the suture of the protoconch (Figure 2, protoconch pointing upwards) by setting landmarks on the larval shell. Morphological analysis of the 2D-slices was carried out using the software IMAGEJ (Schneider et al. 2012). The apical angle (Figure 2A), maximum larval shell height (Figure 2B), maximum larval shell width measured perpendicular to the spire axis (Figure 2C) and adult shell diameter excluding the keel (Figure 2D) were measured from the 2D-slice three times and the means were calculated. Additionally, the number of whorls in the larval shell and the total number of whorls in the shell were counted using the method of Seapy (1990). For statistical analyses of the morphological data, the software PAST v3.12 was used (Hammer et al. 2001). Normal distributions were tested using the Shapiro-Wilk test (Shapiro 1965). To determine the most important variables, a Principal Component Analysis (PCA) was carried out. T-tests were used to detect significant differences between the clades with regards to those informative variables.

Scanning Electron Microscopy (SEM) images were also made on a subset of specimens to investigate the surface shell morphology and ornamentation for each clade. Two specimens of *A. vanderspoeli* and three specimens of *A. turriculata* were scanned using a JEOL JSM-7600F Field Emission SEM. For *A. brunnea*, images of two specimens from Wall-Palmer et al. (2018b) were used.

### Molecular methods

DNA extraction was carried out on whole specimens using the NucleoMag 96 Tissue kit on a Thermo Scientific KingFisher Flex magnetic particle processor with a final elution volume of 75 µl. A ~920 bp fragment of the nuclear 18S rRNA gene was amplified using primers 18S-KP-F (Burridge et al. 2017b) and 1800R (Vonnemann et al. 2005). A ~868 bp fragment of the nuclear 28S rRNA gene was amplified using primers C1-F (Dayrat et al. 2001) and D3-R (Vonnemann et al. 2005). Additionally, a ~658 bp fragment of the mitochondrial cytochrome *c* oxidase subunit 1 gene (mtCO1) (Hebert et al. 2003) was amplified using primers jgLCO1490 and jgHCO2198 (Geller et al. 2013). All primers were tailed with M13F and M13R for Sanger sequencing (Messing 1983). PCR reactions contained 17.3 µl ultrapure water (milliQ), 2.5 µl Qiagen 10× PCR buffer CL, 0.5 µl 25 mM MgCl_2_, 1 µl 10 mg/ml Life BSA, 1.0 µl 10 pMol/µl of each primer, 0.5 µl 2.5 mM dNTPs, 0.25 µl 5U/µl Qiagen Taq and 1 µl of DNA extract (diluted either 5 or 10 times). For all three genes, PCR was carried out using a BIO-RAD Thermal Cycler and starting with an initial denaturation step of 3 min at 96 °C, followed by 40 cycles of denaturation of 15 s at 96 °C, annealing of 30 s at 50 °C, extension of 40 s at 72 °C and a final extension step of 5 min at 72 °C. PCR products were sequenced by BaseClear (Leiden). All sequences are publicly available through BOLD (see Table 1 for accession numbers). Molecular results have been previously published for some of the specimens (Wall-Palmer et al. 2018b, in preparation).

### Phylogenetic analyses

Sequences (forward and reverse strands) were assembled, aligned and verified in Geneious R8 and multiple sequence alignment was performed using MEGA-X (Kumar et al. 2016a). The resulting length of sequence alignments for CO1, 28S and 18S were 658 bp, 868 bp and 920 bp respectively. Phylogenetic relationships were estimated based on single-gene Maximum Likelihood analyses performed in RAxML 8.2.9 (Stamatakis 2014) using the General Time Reversible (GTRCAT) model followed by bootstrap analyses of 1000 replicates (CO1) or 1500 replicates (18S, 28S). Phylogenetic analysis of the three genes combined (2447 bp, GTRCAT, partitioned, 3000 replicates) included) included a subset of 14 specimens (plus three specimens of *A. helicinoidea*), with two specimens per clade per region (in regions where clades are present). All three genes were available for all of the specimens (Table 1). CO1 Genetic distances between and within clades/species were calculated using a Kimura-2-parameter (KP2) substitution model with uniform rates in MEGA-X (Kumar et al. 2016b). Three specimens of *Atlanta helicinoidea* were used as an outgroup in all phylogenetic analyses.

## Results

The phylogenetic, morphological and biogeographical analyses produced a congruent result, distinguishing three species within the *A. brunnea* species group. This includes the two previously known species, *A. brunnea* and *A. turriculata*. In addition, the previously described *A. brunnea* form B, (Wall-Palmer et al. 2018b) also known as *A. turriculata* form B (van der Spoel 1972, 1976) is a valid new species and is formally named here *A. vanderspoeli* sp. nov.

### Phylogenetic analyses

**Figure 3.**
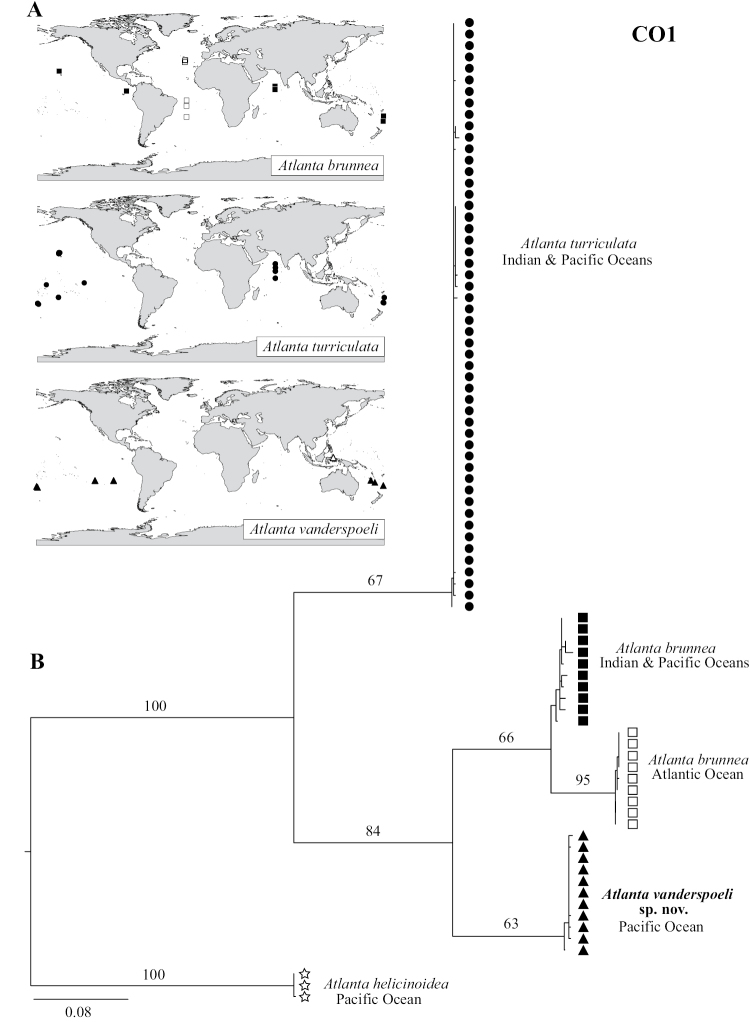
**A** Distribution maps showing the collection locations for each clade identified in B. The collection location of specimens of *A. turriculata* forma B identified by van der Spoel (1976) from offshore of Ternate Island is marked with a white triangle **B** maximum likelihood phylogeny based on the mitochondrial cytochrome *c* oxidase subunit 1 gene, with strong bootstrap support for four clades within the *A. brunnea* group. *Atlanta vanderspoeli* is supported as a valid species, and *A. brunnea* is formed of two geographically isolated clades. Bootstrap support (%) for nodes is displayed and branch lengths are proportional to the amount of inferred change, as indicated by the scale bar (mean number of nucleotide substitutions per site).

**Figure 4.**
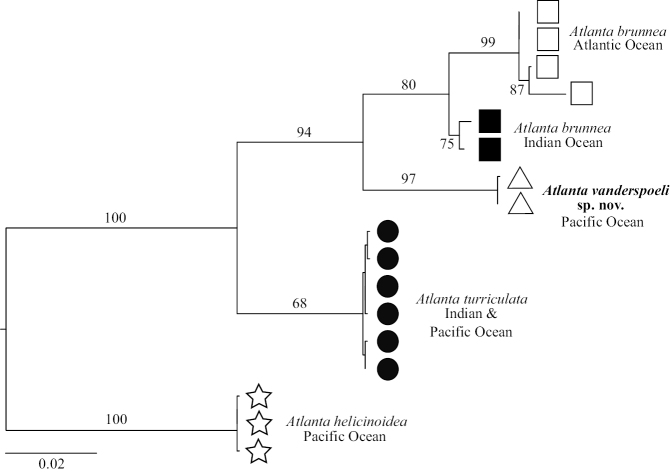
Maximum likelihood phylogeny of the *A. brunnea* species group based on analysis of the combined genes CO1, 28S and 18S with a total alignment of 2447 bp. All four clades within the *A. brunnea* species group are monophyletic with strong bootstrap support. Bootstrap support (%) for nodes is displayed and branch lengths are proportional to the amount of inferred change, as indicated by the scale bar (mean number of nucleotide substitutions per site).

Phylogenetic analyses of CO1 (Figure 3) and combined genes (Figure 4) recover these three species with moderate to high bootstrap support. The single gene phylogenies of 18S and 28S resolve deeper relationships, but do not resolve the more recent divergences at the species level within the *A. brunnea* species group (Suppl. material 1: Figures S1–S2). All of the single and combined gene phylogenetic analyses support the *A. brunnea* species group as being monophyletic with 100 % node support. In the combined gene phylogeny (Figure 4), *A. vanderspoeli* forms a monophyletic group with 97% node support, supporting inference that this is a valid species. *Atlanta brunnea* also forms a well-supported (80 % for the combined genes) monophyletic group, but this is further split into two clades. *Atlanta turriculata* forms a monophyletic group (Indian and Pacific Oceans), but this is only moderately supported (68% node support).

Genetic distances for CO1 support the position of *A. vanderspoeli* as a valid new species. *Atlanta brunnea* and *A. vanderspoeli* are separated by a CO1 genetic distance of 13–16% (Table 2). These genetic distances are comparable to the CO1 genetic distances between the previously described species within this species group, *A. brunnea* and *A. turriculata* (18–20 %). CO1 genetic distances between *A. brunnea*, *A. vanderspoeli*, *A. turriculata* and the outgroup species *A. helicinoidea* are 22–25%, 23–24% and 21–22%, respectively (Table 2).

**Table 2.** Mean values (in bold) and ranges for genetic distances among members of the *A. brunnea* species group, based on a 658 bp fragment of the mitochondrial cytochrome *c* oxidase subunit 1 gene calculated using a Kimura 2-parameter substitution model with uniform rates.

**CO1**	***A. brunnea* Atlantic**	***A. brunnea* Indian & Pacific**	***A. vanderspoeli* Pacific**	***A. turriculata* Indian & Pacific**	***A. helicinoidea* Pacific**
*A. brunnea* Atlantic	**0**				
	0				
*A. brunnea* Indian & Pacific	**6**	**1**			
	6–7	0–1			
*A. vanderspoeli* Pacific	**16**	**14**	**0**		
	15 –16	13–15	0–1		
*A. turriculata* Indian & Pacific	**2**	**19**	**19**	**0**	
	19–20	18–20	18–20	0–1	
*A. helicinoidea* Pacific	**24**	**23**	**24**	**21**	**0**
	24–25	22–24	23–24	21–22	0

Within *A. brunnea*, the molecular data identify a further separation into two well supported geographic clades, one containing the Indian and Pacific Ocean population and one containing the Atlantic Ocean population (Figure 4, node support of 75% and 99% respectively for the combined genes). There is a CO1 genetic distance of 6–7% between specimens from the Atlantic Ocean and specimens from the Indian and Pacific oceans. This genetic distance is less than distances between other species within the *A. brunnea* group, and therefore, one of these two populations is likely to be an incipient species. However, the type locality of *A. brunnea* is not known for certain, Gray (1850) having written only ‘*A. brunnea*, Eydoux’ to accompany his drawing of this species. Here we accept the lectotype locality because we assume that Gray illustrated the same specimens that Souleyet (1852) named *A. fusca* (originally given the vernacular name “Atlanta brune”), as they both originated from specimens collected by *Eydoux* on the “Bonite” expedition and the illustrations are almost identical (Suppl. material 1: Figure S3). The type locality for *A. fusca* is the Indian Ocean and so we assume here that the type locality of *A. brunnea* is also the Indian Ocean. Therefore, the newly detected Atlantic Ocean population should be considered as the incipient species.

### Shell morphology

**Figure 5.**
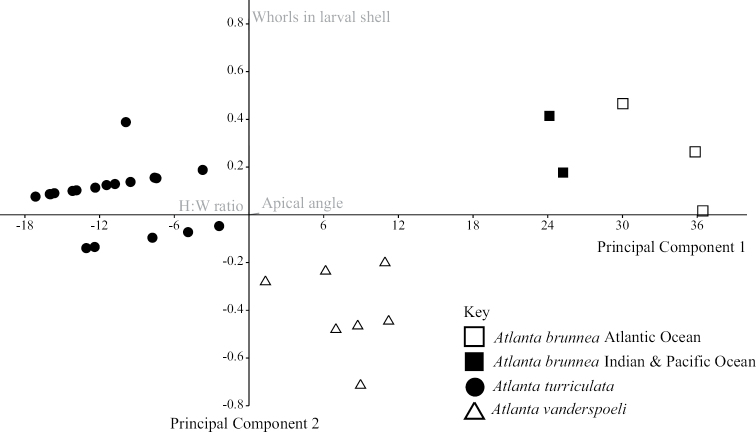
Principal Component Analysis (PCA) performed on apical angle, height: width ratio and the number of whorls in the larval shell. Species identity confirmed for most specimens (*N* = 25) using DNA barcoding (CO1). Two specimens of each species (total *N* = 6) derive from the DANA collection, and could not be DNA barcoded (formalin-fixed). Morphometric data are reported in Suppl. material 2:Table S1.

**Figure 6.**
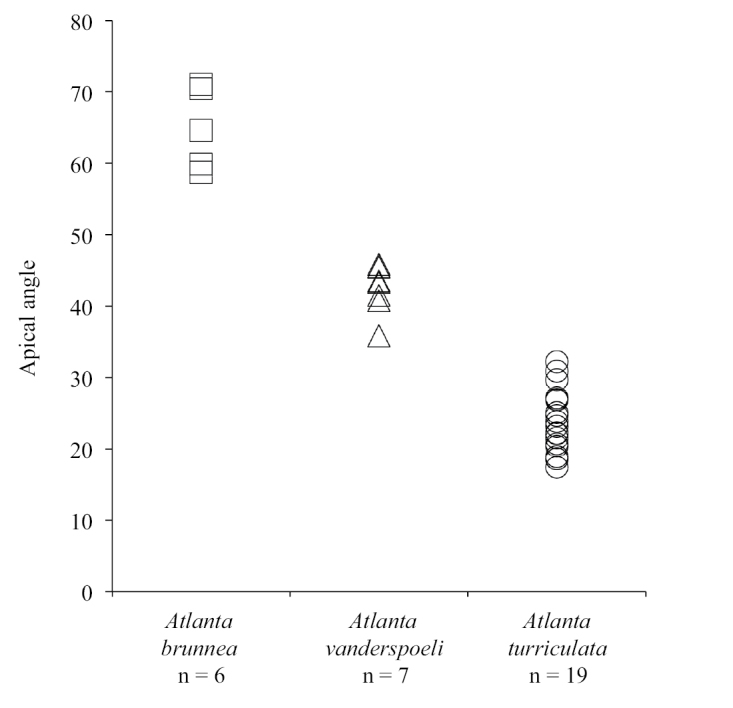
Shell apical angles of *A. brunnea* (Atlantic, Pacific and Indian oceans), *A. vanderspoeli* (Pacific Ocean) and *A. turriculata* (Pacific and Indian oceans) are significantly different and do not overlap.

Principal Component Analysis (PCA) identifies the apical angle of the protoconch and the number of whorls in the larval shell as the most informative morphological characters to distinguish between *A. brunnea*, *A. vanderspoeli*, and *A. turriculata* (Figure 5). The apical angle of *A. brunnea* is the widest (58.7°–71.0°), followed by *A. vanderspoeli* (35.9°–45.8°) and *A. turriculata* (17.4°–32.2°, Suppl. material 2: Table S1, Figure 6). The difference in apical angle between *A. brunnea* and *A. vanderspoeli*, and between *A. turriculata* and *A. vanderspoeli* is significant (t-test, p=<0.001 for both; Figure 6). Differences in the number of whorls in the larval shell are also identified by the data. *Atlanta brunnea* has 3.75–4.25 whorls, *A. vanderspoeli* has 3.25–4.00 whorls and *A. turriculata* has 4.00–4.50 whorls in the larval shell (Suppl. material 2: Table S1). The height to width ratio of the larval shell, maximum number of whorls (in the whole shell) and adult shell diameter overlap for the three clades and cannot be used as reliable identifying features (Suppl. material 2: Table S1). The low number of specimens does not allow an assessment of morphological differences between the Atlantic Ocean and the Pacific and Indian Ocean specimens of *A. brunnea*.

**Figure 7.**
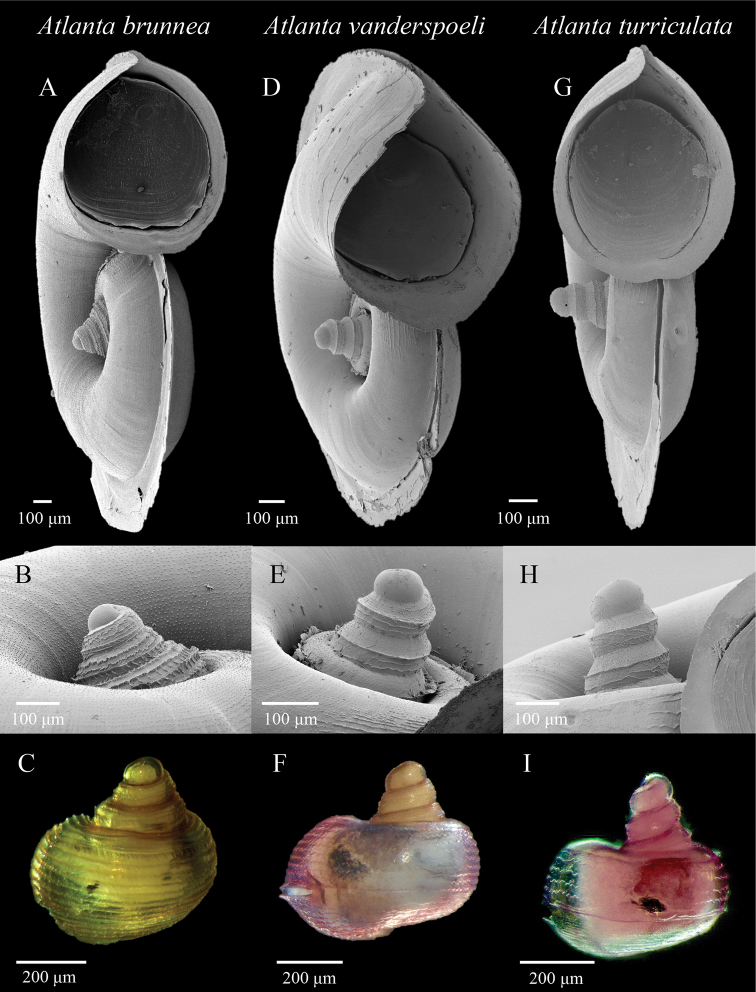
Scanning Electron Microscopy (SEM) and stacked light microscopy images of representative specimens of *A. brunnea*: **A, B** DANA_3929VIII **C** SN105_08, *A. vanderspoeli*: **D–E** DANA_3558VII (Holotype, NHMD-232132) **F** KH1110_15 and *A. turriculata*: **G, H** DANA_3929VIII **I** SN105_19. Apical angle is the most useful morphological feature for distinguishing between the species (**B–C, E–F, H–I**). The shell of *A. brunnea* is always brown (**C**); however, the colour of *A. vanderspoeli* and *A. turriculata* shell and soft tissues (**F, I**) can vary and these are not reliable features for identification.

**Figure 8.**
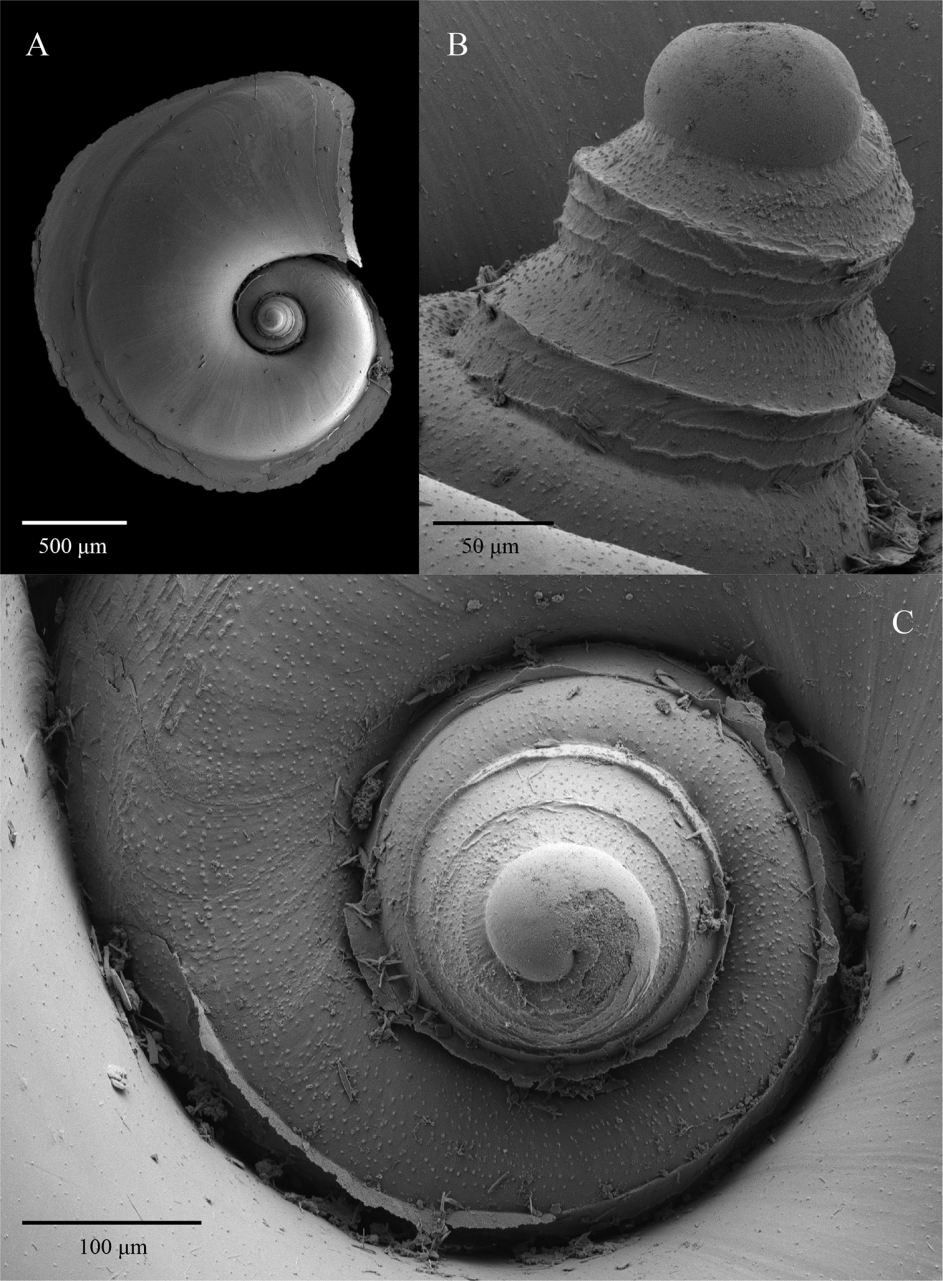
SEM images of the *A. vanderspoeli* holotype from station DANA_3558VII (NHMD-232132) **A** apical view of the entire shell showing the rapid inflation of the shell **B** magnified view of the micro-ornamentation **C** magnified apical view showing the extent of the carina.

Ornamentation is limited in its use as an identifying feature for this species group, and the fine micro-ornamentation is only really visible with the use of SEM. All three species have a prominent ridge, or carina that runs slightly above mid-whorl height of the larval shell. The larval shell of *A. brunnea* is easily identified by the heavy zig-zag, and in places, net-like reticulate spiral lines covering the surface (Figure 7B). However, the ornamentation of *A. vanderspoeli* is more difficult to tell apart from *A. turriculata*. In both, the larval shell is more sparsely ornamented than in *A. brunnea*. In *A. vanderspoeli*, interrupted spiral lines and small projections roughly arranged in lines are found above the carina of each whorl, and zig-zag ornamentation is found below the carina of each whorl (Figure 7E, Figure 8). In *A. turriculata*, there is generally little or no ornamentation above the carina of each whorl, but in common with the other two species, zig-zag ornamentation is found below it (Figure 7H).

### Biogeography

Data gathered in this study expand the known distribution of the *A. brunnea* group (Wall-Palmer et al. 2016, 2018b). *Atlanta brunnea* was found to have the broadest distribution, in the Atlantic Ocean (north and south subtropical gyres), in the Indian Ocean and in the Pacific Ocean (Figure 3). *Atlanta brunnea* was not found in the central southern Pacific Ocean, although this is based upon limited sampling coverage in this region. Conversely, *A. vanderspoeli* was only found in the equatorial and southern Pacific Ocean (0.80N–29.95S, 127.28E–100.00W). The two species only overlap in the south west and the east Pacific Ocean (Figure 3). The molecular results presented here show that the Atlantic Ocean population of *A. brunnea* is a geographically divergent species. *Atlanta turriculata* was found throughout the Pacific and Indian oceans, overlapping with the distribution of *A. vanderspoeli* in the equatorial and South Pacific Ocean. In agreement with previous studies, *A. turriculata* was not found in the Atlantic Ocean (Figure 3).

## Discussion

### A note on *Atlanta turriculata* d’Orbigny, 1836

**Figure 9.**
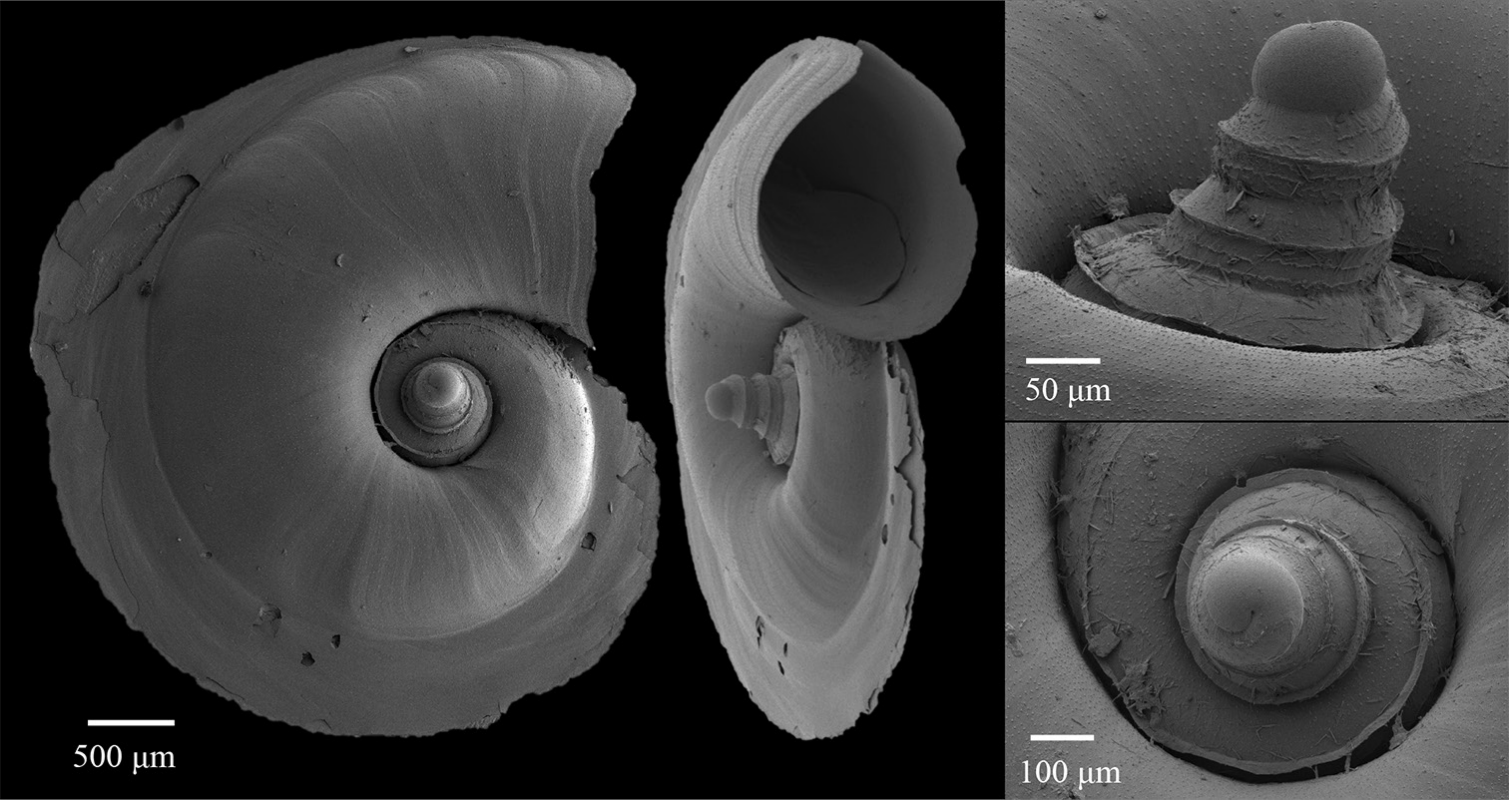
*Atlanta turriculata* neotype RMNH.MOL.342213, housed at the Naturalis Biodiversity Center.

*Atlanta turriculata* was described by d’Orbigny (1836) from the South Pacific Ocean. This is the region in which the distributions of *A. turriculata* and *A. vanderspoeli* overlap and so it is necessary to check that d’Orbigny described and illustrated specimens of *A. turriculata* and not the morphologically similar *A. vanderspoeli*. Unfortunately, type material of *A. turriculata* could not be found at either the Natural History Museum, London (NHMUK), or the Muséum National d’Histoire Naturelle, Paris (MNHN), where it should be present (syntype material located at NHMUK was discovered to be mislabeled specimens of *Oxygyrus*). In addition, the illustrations of *A. turriculata* made by d’Orbigny are not sufficiently detailed to determine which species he actually drew. Although the more widely geographically distributed, narrow-turreted spired species is now well known as *A. turriculata*, we find it necessary to solve this potential confusion by depositing a neotype specimen of (what is now widely known as) *A. turriculata* (see Systematics section for details, Figure 9).

### The validity of *A. vanderspoeli* sp. nov.

**Figure 10.**
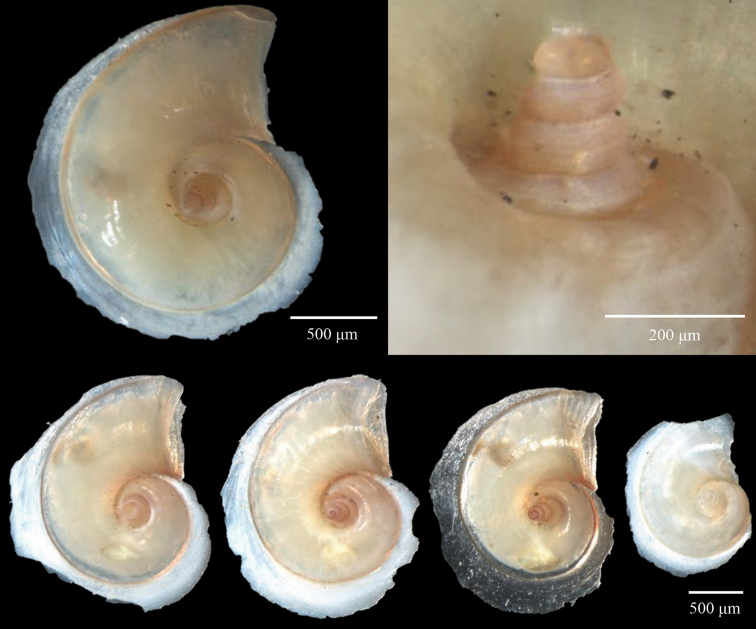
Five specimens of *Atlanta turriculata* forma B identified by van der Spoel and held in the collection at the Naturalis Biodiversity Center. These specimens are now designated as paratypes of *Atlanta vanderspoeli* RMNH.MOL.342212.

Additional diversity within the *A. brunnea* species group has long been recognised, but, until now, this has not been thoroughly investigated. Van der Spoel (1972) first noticed *A. vanderspoeli* when he remarked on an atlantid form similar to *A. turriculata*, calling it *A. turriculata* forma B. Initially recognised from the soft tissues, van der Spoel described *A. turriculata* forma B as generally having a lack of tubercules on the operculum, which were present in *A. turriculata*. Later, van der Spoel (1976: 146–147) described the shell as having ‘whorls in the spire [that] increase more rapidly in width so that the spire in relation to the body whorl is larger than in [*A. turriculata*]’. Van der Spoel deposited specimens of this forma, which are now in the collection of the Naturalis Biodiversity Center, Leiden (Figure 10). More recently, Wall-Palmer et al. (2018b) detected an additional clade within the *A. brunnea* group using a CO1 phylogeny of a global collection of specimens. This clade was more closely related to *A. brunnea*, and was referred to as *A. brunnea* form B. Here we considered three species concepts to determine whether this new clade, referred to in this study as *A. vanderspoeli*, was a valid species; the morphological species concept, the biological isolation (biogeographical) species concept and the phylogenetic species concept (De Queiroz 2007). Under each of these concepts, our results verify that *A. vanderspoeli* is different from the two most closely related species, *A. brunnea* and *A. turriculata*. Although there were only a relatively small number of specimens available for morphological analysis, *A. vanderspoeli* shells were found to be morphologically distinct, having an apical angle that does not overlap with its two closest relatives. The combined three gene phylogeny supports *A. vanderspoeli* to be genetically distinct from *A. brunnea* and *A. turriculata*, with genetic distances comparable to other species within the group. *Atlanta vanderspoeli* also appears to have a relatively restricted distribution, only being found (thus far) in the equatorial and South Pacific Ocean. The pteropod species *Cuvierina tsudai* Burridge, Janssen & Peijnenburg, 2016 has a very similar geographical distribution (Burridge et al. 2015, 2016). The known distribution of *A. vanderspoeli* only overlaps with *A. brunnea* and *A. turriculata* at the very edges of their populations. Relatively poor spatial resolution of molecular data in the Pacific Ocean means it is possible that the distribution of *A. vanderspoeli* is also wider (e.g. in the north Pacific). The species is therefore not strongly geographically isolated from its two most closely related species. However, these results are compatible with the phylogenetic species concept, because even though the species overlap geographically and likely do meet in nature, there must be no interbreeding as evidenced by the combined gene phylogeny (and 28S, Suppl. material 1: Figure S1).

### An Atlantic Ocean incipient species

Results of the phylogenetic analysis also highlight a probable incipient species. The morphospecies *A. brunnea* was found to have two genetically different populations, one in the Indian and Pacific Oceans, and one in the Atlantic Ocean. The genetic distance between the two populations is relatively small, but suggests that the populations of *A. brunnea* in the Atlantic Ocean must be isolated and becoming a new species. This result agrees with Wall-Palmer et al. (2018b) who suggested the existence of an additional genetic lineage but due to insufficient data this could not be confirmed as a distinct species. Richter (1961) also noted differences in the Atlantic population of *A. fusca* (= *A. brunnea*), describing them as having smaller, more pigmented shells when compared to specimens in the Indian Ocean. Differences in the operculum and radula were also reported by Richter (1961). We did not detect any morphological differences between the two populations of *A. brunnea*, however, only shell ornamentation and the measurements of the larval shell have been analysed here.

## Systematics

### Phylum Mollusca

#### Class Gastropoda Cuvier, 1797

**Subclass Caenogastropoda Cox, 1960**

**Order Littorinimorpha Golikov & Starobogatov, 1975**

**Superfamily Pterotracheoidea Rafinesque, 1814**

**Family Atlantidae Rang, 1829**

**Genus *Atlanta* Lesueur, 1817**

##### Atlanta vanderspoeli

Taxon classificationAnimaliaLittorinimorphaAtlantidae

Wall-Palmer, Hegmann & Peijnenburg sp. nov.

8DE0CE00-C2AB-5A26-A0B5-736C4E942AE4

http://zoobank.org/10CC5F39-3E0A-4A53-B7D3-74F4DA5BA966

Figures 7 , 8

Atlanta turriculata forma B – van der Spoel 1972: 550
Atlanta turriculata forma B – van der Spoel 1976: 146–147
Atlanta brunnea – Quesquén Liza 2017: 265
Atlanta brunnea form B – Wall-Palmer et al. 2018b: 10, Fig. 3

###### Type locality.

DANA expedition (1928–1930) station 3558VII, South Pacific 0.30S, 99.12W. Specimen collected on the 18^h^ September 1928 at 19:00 from 200–300 m water depth.

***Holotype*.** Figure 7D–E, NHMD-232132, Housed at the Natural History Museum of Denmark, Copenhagen.

***Paratypes*.**NHMD-232153. DANA_3613V, South Pacific 22.72S, 166.10E. Specimen collected on 28 April 1928 at 03:15 from 100–200 m water depth. Housed at the Natural History Museum of Denmark, Copenhagen. RMNH.MOL.342212. Type material of *A. turriculata* forma B (Figure 10) was deposited by van der Spoel in the collections of the Zoological Museum of Amsterdam (now housed at the Naturalis Biodiversity Center, Leiden, RMNH). We designate these specimens as paratypes of *A. vanderspoeli*. They were collected during the “Siboga” Expedition at station 136 in the equatorial Pacific 0.80N, 127.28E.

###### Diagnosis.

*Atlanta* species with a larval shell of 3 ¼ to 4 whorls. The larval shell is much higher than wide, conical with an apical angle of 35–46°. The larval shell has a prominent carina slightly above mid-whorl height. The whorls of the larval shell are further covered in a micro-ornamentation of interrupted spiral lines and small projections roughly arranged in spiral lines (approximately five lines in total) above this carina, and zig-zagged ornamentation below it (Figs 7D–F, 8).

###### Description.

The shell is small and fresh specimens vary in colour from brown to pink-purple. The adult shell is on average 1000 μm in diameter without the keel (Suppl. material 2: Table S1). The shell begins to inflate on the boundary of teleoconch and protoconch at 3¼ to 4 whorls and has a total of 4¾ to 5 whorls in adults. The keel begins after 3¾ to 4 whorls and inserts between the final whorl and the preceding in larger specimens (filling the space between the whorls). The keel is tall (~400 μm), thin and transparent, and gradually truncates towards the aperture (after ca. ¾ of a whorl). The soft tissues of the foot/fin and sucker can be mottled black (see Suppl. material 3: video clip). The last whorl of the larval shell is ca. 262 to 372 μm in diameter (Suppl. material 2: Table S1). The larval shell is high and conical, with heavy ornamentation covering the surface. A prominent carina is situated slightly above mid-whorl height and is visible with light microscopy. The spiral lines and zig-zag ornamentation is more clearly visible using SEM. The operculum is type a (macro-oligogyre) and the eyes are of type a, with no transverse slit (Seapy et al. 2003).

###### Discussion.

The shape of the larval shell, shell size, colouration, operculum and eye type demonstrate that *A. vanderspoeli* belongs within the *A. brunnea* species group. This is supported by the molecular analysis presented within this study and by Wall-Palmer et al. (2018b). Molecular data finds *A. vanderspoeli* to be most closely related to *A. brunnea*, followed by *A. turriculata*. These are also morphologically the most similar species. *Atlanta vanderspoeli* can be distinguished from these two species using the apical angle, which is more narrow than in *A. brunnea*, but wider than in *A. turriculata*. Van der Spoel (1972) noted that the operculum of (*A. turriculata* forma B) *A. vanderspoeli* generally does not have tubercles/spines, or that these are less developed (van der Spoel 1976) compared to the operculum of *A. turriculata*. Due to a lack of adult specimens, we were unable to extract an operculum to confirm this.

###### Distribution.

All specimens were found in the equatorial and south Pacific Ocean from 0.80N to 29.95S, and from 127.28E to 100.00W (Figure 3). Specimens were collected from the upper 600 m using oblique and vertical plankton tows. During this study we only found specimens of *A. vanderspoeli* in the south Pacific Ocean. However, specimens identified as *A. turriculata* forma B by van der Spoel (1976) were collected at ‘Ternate Anchorage’ in the Indomalayan Archipelago (“Siboga” Expedition station 136, equatorial Pacific 0.80N, 127.28E. Figure 10).

###### Etymology.

Named after Professor emeritus Siebrecht van der Spoel, who first noticed *A. vanderspoeli*, but described it only as *A. turriculata* forma B due to a lack of specimens (van der Spoel 1976). Professor van der Spoel spent many years working on holoplanktonic gastropods and made important contributions to our understanding of their taxonomy and distributions.

##### Atlanta turriculata

Taxon classificationAnimaliaLittorinimorphaAtlantidae

d’Orbigny, 1836

03AED2CE-2ED8-5888-8B4A-4E918BDEAC9D

Figure 9

###### Original type locality.

The great Austral Ocean between 30 and 34S.

***Holotype*.** We have been unable to locate any type material for *A. turriculata* (thought to have been deposited in NHMUK or MNHN).

***Neotype*.** RMNH.MOL.342213. SN105_08, Indian Ocean 4.38N, 67.00E. Specimen collected during the SN105 expedition of the ORV *Sagar Nidhi*, on the 10^th^ December 2015 at 04:40 from 67 m water depth. Housed at the Naturalis Biodiversity Center, Leiden.

Two additional specimens have been deposited at the Natural History Museum, London. NHMUK20191155 and NHMUK20191156, from station KOK1703_06, Pacific Ocean 23.52N, 156.77W. Specimens collected during the KOK1703 expedition of the RV “Ka’Imikai-O-Kanaloa” on the 3^rd^ September 2017 at 03:59 from 0–250 m water depth.

The holotype of *Atlanta vanderspoeli* is the property of the Natural History Museum of Denmark, Copenhagen. The neotype of *Atlanta turriculata* is the property of the Naturalis Biodiversity Center, Leiden. Both institutes maintain a research collection with proper facilities for preserving name-bearing types and these types are made accessible for study.

## Conclusions

Using an integrated species concept, this study demonstrates that the *A. brunnea* group contains three valid species, *A. brunnea*, *A. turriculata*, and *A. vanderspoeli* sp. nov. A further incipient species that is restricted to the Atlantic Ocean should also be considered in future research, in particular during ecological or experimental studies. We hope that the integrated approach to species validation reported here will facilitate other workers in identifying *A. vanderspoeli* in their studies. Increased spatial coverage is now needed to fully understand the current distributions and environmental tolerances of this new species. Only with a larger and more complete dataset, including collection depths and concurrently collected environmental data, will it be possible to understand the responses of these atlantids to a changing ocean.

## Supplementary Material

XML Treatment for Atlanta vanderspoeli

XML Treatment for Atlanta turriculata

## Supplementary materials

Supplementary material 1 Figures S1–S3 Data type: multimedia Explanation note: **Figure S1.** Maximum likelihood phylogeny of the *A. brunnea* group based on 28S. **Figure S2.** Maximum likelihood phylogeny of the *A. brunnea* group based on 18S. **Figure S3.** The original illustration of (**A**) *Atlanta brunnea* by Gray (1850) is clearly copied from the original illustration by Eydoux and Souleyet (1841) of (**B**) *Atlanta brune* (vernacular name) later formally named *A. fusca* (Souleyet, 1852). https://binary.pensoft.net/file/364536This dataset is made available under the Open Database License (http://opendatacommons.org/licenses/odbl/1.0/). The Open Database License (ODbL) is a license agreement intended to allow users to freely share, modify, and use this Dataset while maintaining this same freedom for others, provided that the original source and author(s) are credited.Deborah Wall-Palmer, Mona Hegmann, Erica Goetze3 Katja T.C.A. Peijnenburg

Supplementary material 2 Table S1. Morphometric data for *A. brunnea*, *A. vanderspoeli* and *A. turriculata* Data type: measurement https://binary.pensoft.net/file/364537This dataset is made available under the Open Database License (http://opendatacommons.org/licenses/odbl/1.0/). The Open Database License (ODbL) is a license agreement intended to allow users to freely share, modify, and use this Dataset while maintaining this same freedom for others, provided that the original source and author(s) are credited.Deborah Wall-Palmer, Mona Hegmann, Erica Goetze3 Katja T.C.A. Peijnenburg

Supplementary material 3 *Atlanta vanderspoeli* video clip Data type: multimedia Explanation note: *Atlanta vanderspoeli* specimen (male) collected during cruise SO255 station 80 at 29.10S, 179.72W. https://binary.pensoft.net/file/364538This dataset is made available under the Open Database License (http://opendatacommons.org/licenses/odbl/1.0/). The Open Database License (ODbL) is a license agreement intended to allow users to freely share, modify, and use this Dataset while maintaining this same freedom for others, provided that the original source and author(s) are credited.Deborah Wall-Palmer, Mona Hegmann, Erica Goetze3 Katja T.C.A. Peijnenburg
