# Supplementary figures and images for: Resolving species boundaries in the Atlanta brunnea species group (Gastropoda, Pterotracheoidea)
Source: Zookeys. 2019 Dec 12;899:59–84. doi: 10.3897/zookeys.899.38892 (PMC6923281; doi:10.3897/zookeys.899.38892)

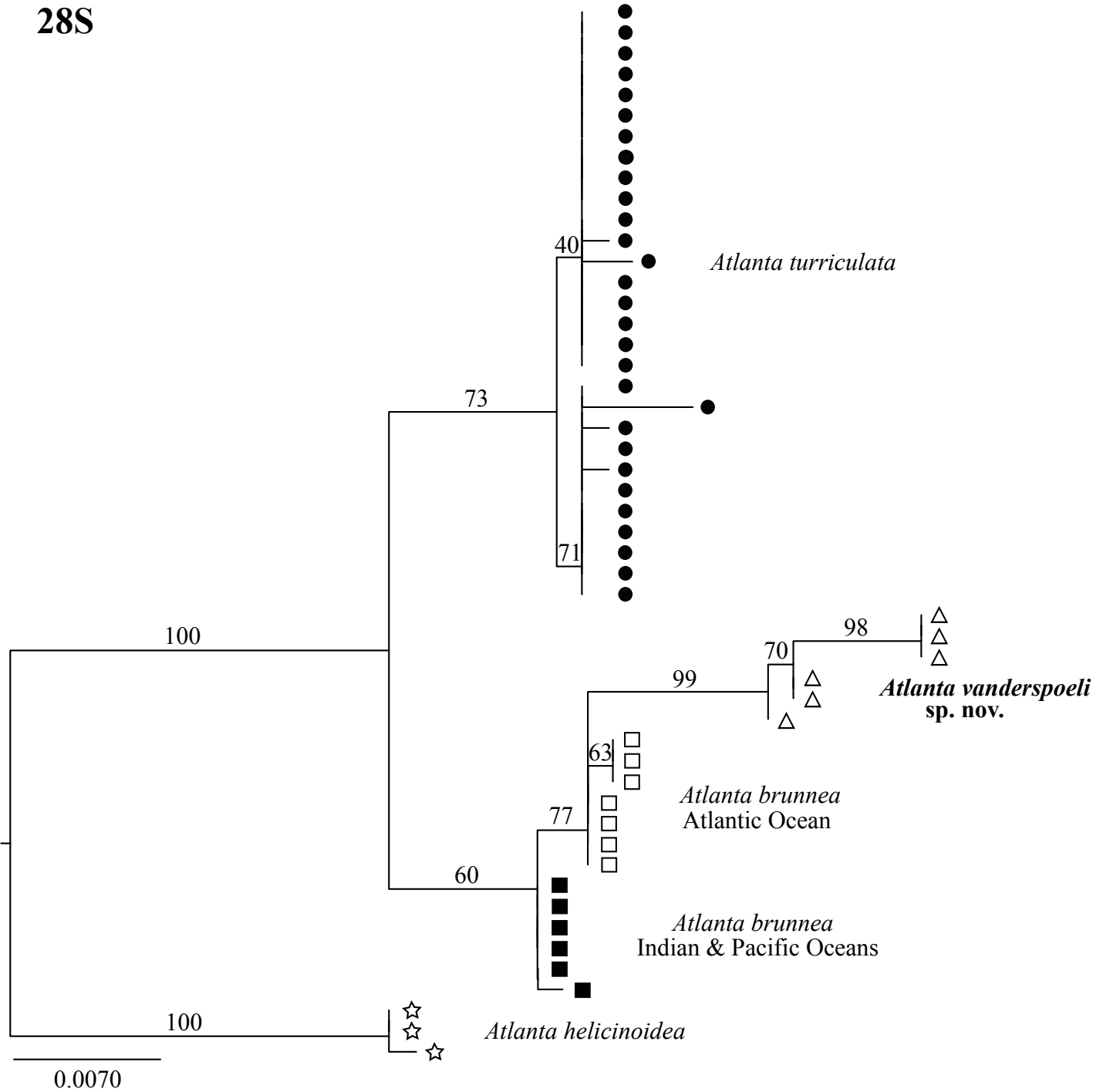

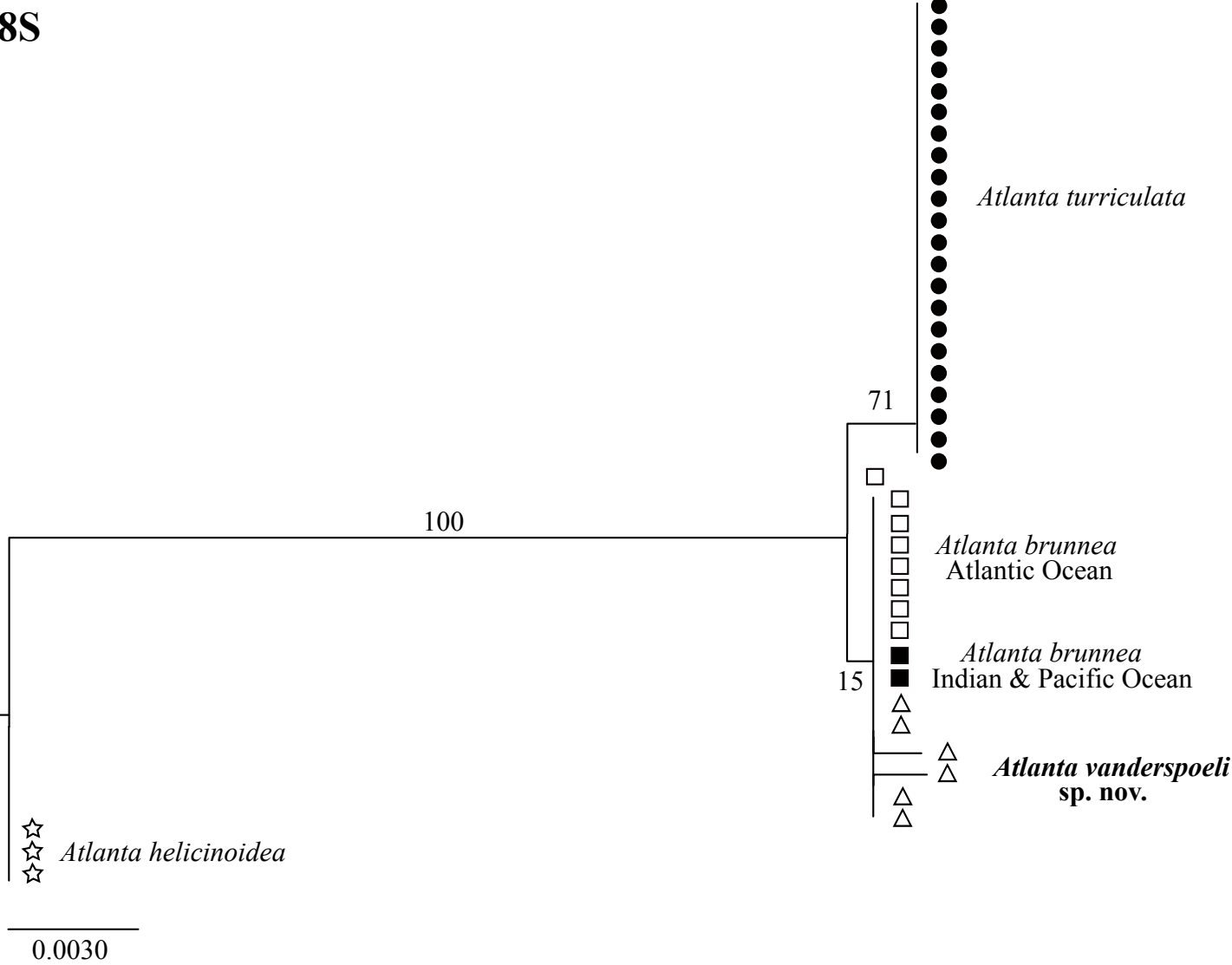

**A**

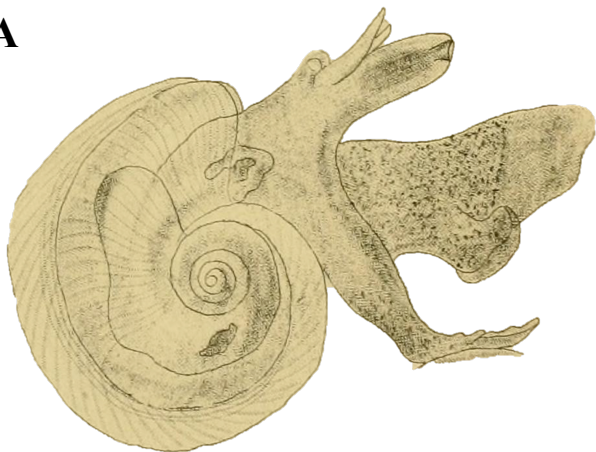

**B**

15

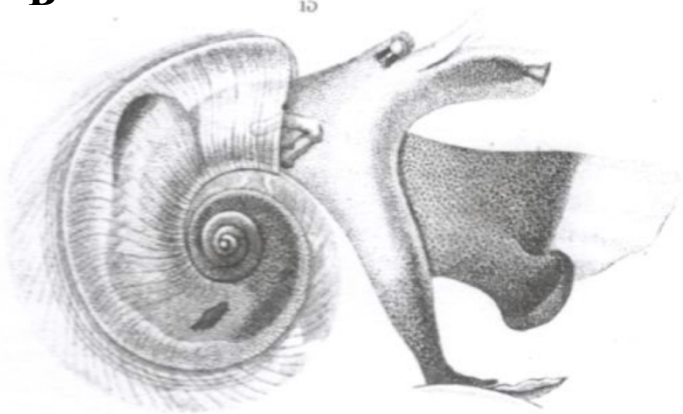

Supplement: Supplementary material 1 [file zookeys-899-059-s001.pdf]
