# Supplementary material for: Resolving species boundaries in the Atlanta brunnea species group (Gastropoda, Pterotracheoidea)
Source: Zookeys. 2019 Dec 12;899:59–84. doi: 10.3897/zookeys.899.38892 (PMC6923281; doi:10.3897/zookeys.899.38892)
Supplement: Supplementary material 2 [file zookeys-899-059-s002.doc]

Supplementary Table 1. Morphometric data for *A. brunnea, A. vanderspoeli* and *A*. *turriculata.* RMNH = Naturalis Biodiversity Center, NHMD = Natural History Museum Denmark. All accession numbers relate to virtual specimens (images) because specimens were destroyed during DNA extraction. NHMD-232153 is a paratype of *A. vanderspoeli* and the specimen remains.

| BOLD accession number | Museum accession number | Cruise_station | Species | Max. larval shell height (µm) | Max. larval shell width (µm) | height: width ratio | Adult shell diameter (µm) | Apical angle | No. whorls in larval shell | No. whorls in adult shell |
| --- | --- | --- | --- | --- | --- | --- | --- | --- | --- | --- |
|
| ATCP009-19 | RMNH.MOL.341315 | AMT27_11 | *Atlanta brunnea* | 522 | 452 | 1.16 | 1467 | 59 | 4.25 | 5.00 |
| ATCP010-19 | RMNH.MOL.341316 | AMT27_11 | *Atlanta brunnea* | 428 | 302 | 1.42 | 954 | 60 | 4.00 | 5.00 |
| - | NHMD-232129 | DANA_3556 VIII | *Atlanta brunnea* | 460 | 356 | 1.29 | 1079 | 70 | 4.00 | 4.50 |
| - | NHMD-232129 | DANA_3556 VIII | *Atlanta brunnea* | 445 | 418 | 1.07 | 1334 | 71 | 3.75 | 4.50 |
| AGD012-17 | RMNH.MOL.341302 | SN105_08 | *Atlanta brunnea* | 464 | 356 | 1.30 |  | 65 | 4.25 | 5.00 |
| - | NHMD-232153 *P | DANA_3613 V | *Atlanta vanderspoeli* | 430 | 290 | 1.48 | 1019 | 44 | 3.25 | 4.50 |
| - | NHMD-232154 | DANA_3620 IV | *Atlanta vanderspoeli* | 380 | 262 | 1.45 | 898 | 46 | 3.50 | 4.75 |
| ATCP012-19 | RMNH.MOL.341319 | SO255_57 | *Atlanta vanderspoeli* | 431 | 282 | 1.53 | 845 | 43 | 3.50 | 4.50 |
| ATCP015-19 | RMNH.MOL.341327 | SO255_73 | *Atlanta vanderspoeli* | 417 | 277 | 1.51 | 971 | 42 | 3.50 | 4.25 |
| - | - | SO255_73 | *Atlanta vanderspoeli* | 446 | 372 | 1.20 | 1355 | 45 | 3.75 | 4.50 |
| ATCP016-19 | RMNH.MOL.341328 | SO255_80 | *Atlanta vanderspoeli* | 416 | 330 | 1.26 | 1242 | 36 | 3.75 | 4.50 |
| ATCP017-19 | RMNH.MOL.341329 | SO255_80 | *Atlanta vanderspoeli* | 430 | 267 | 1.61 | 948 | 41 | 3.75 | 4.50 |
| - | NHMD-232140 | DANA_3563 IV | *Atlanta turriculata* | 508 | 378 | 1.35 | 1523 | 27 | 4.25 | 5.00 |
| - | NHMD-232145 | DANA_3586 VII | *Atlanta turriculata* | 461 | 231 | 2.00 | 1329 | 32 | 4.00 | 5.00 |
| ATCP112-19 | RMNH.MOL.341803 | KOK1703_01 | *Atlanta turriculata* | 522 | 319 | 1.64 | 847 | 22 | 4.00 | 4.75 |
| ATCP116-19 | RMNH.MOL.341807 | KOK1703_03 | *Atlanta turriculata* | 362 | 247 | 1.47 | 912 | 22 | 4.25 | 5.00 |
| ATCP118-19 | RMNH.MOL.341809 | KOK1703_03 | *Atlanta turriculata* | 400 | 244 | 1.64 | 756 | 27 | 4.00 | 4.25 |
| ATCP120-19 | RMNH.MOL.341811 | KOK1703_03 | *Atlanta turriculata* | 518 | 346 | 1.50 | 1346 | 25 | 4.25 | 5.25 |
| ATCP122-19 | RMNH.MOL.341813 | KOK1703_03 | *Atlanta turriculata* | 476 | 262 | 1.82 | 946 | 25 | 4.50 | 4.75 |
| ATCP124-19 | RMNH.MOL.341815 | KOK1703_03 | *Atlanta turriculata* | 492 | 287 | 1.72 | 1091 | 21 | 4.00 | 4.75 |
| ATCP125-19 | RMNH.MOL.341816 | KOK1703_05 | *Atlanta turriculata* | 463 | 259 | 1.78 | 841 | 19 | 4.25 | 4.50 |
| ATCP127-19 | RMNH.MOL.341818 | KOK1703_05 | *Atlanta turriculata* | 494 | 310 | 1.59 | 1189 | 30 | 4.00 | 5.00 |
| ATCP129-19 | RMNH.MOL.341820 | KOK1703_05 | *Atlanta turriculata* | 413 | 257 | 1.61 | 1007 | 20 | 4.25 | 5.00 |
| ATCP113-19 | RMNH.MOL.341804 | KOK1703_06 | *Atlanta turriculata* | 410 | 256 | 1.60 | 893 | 21 | 4.25 | 4.75 |
| ATCP117-19 | RMNH.MOL.341808 | KOK1703_06 | *Atlanta turriculata* | 461 | 275 | 1.68 | 855 | 19 | 4.25 | 4.75 |
| ATCP119-19 | RMNH.MOL.341810 | KOK1703_07 | *Atlanta turriculata* | 487 | 295 | 1.65 | 549 | 24 | 4.25 | 4.50 |
| ATCP121-19 | RMNH.MOL.341812 | KOK1703_08 | *Atlanta turriculata* | 455 | 260 | 1.75 | 1062 | 23 | 4.25 | 4.50 |
| ATCP123-19 | RMNH.MOL.341814 | SN105_08 | *Atlanta turriculata* | 457 | 270 | 1.70 | 1027 | 17 | 4.25 | 5.00 |
| - | - | SO255_01 | *Atlanta turriculata* | 435 | 236 | 1.85 | 767 | 19 | 4.25 | 4.50 |
| ATCP126-19 | RMNH.MOL.341817 | SO255_73 | *Atlanta turriculata* | 535 | 325 | 1.65 | 1215 | 27 | 4.25 | 5.00 |
| ATCP128-19 | RMNH.MOL.341819 | SO255_73 | *Atlanta turriculata* | 458 | 275 | 1.67 | 944 | 31 | 4.25 | 5.00 |
